# Adjuvant melatonin therapy during exercise prescription in breast cancer survivors on physical and anthropometric parameters, quality of life, and hormonal response. A randomized controlled trial

**DOI:** 10.3389/fspor.2025.1594733

**Published:** 2025-06-23

**Authors:** Ana M. Celorrio San Miguel, Luis M. Cacharro, Gema Santamaría, Manuel Garrosa, Marta Celorrio San Miguel, Enrique Roche, Evelina Garrosa, Diego Fernández-Lázaro

**Affiliations:** ^1^ Doctoral School, University of Leon, Leon, Spain; ^2^ Department of Ophthalmology of Salamanca University Assistance Complex (CAUSA), Salamanca University Hospital, Salamanca, Spain; ^3^ Department of Anatomy and Radiology, Faculty of Health Sciences, University of Valladolid, Soria, Spain; ^4^ Area of Histology, Faculty of Medicine, Institute of Neurosciences of Castile and Leon (INCYL), University of Valladolid, Valladolid, Spain; ^5^ Neurobiology Research Group, Faculty of Medicine, University of Valladolid, Valladolid, Spain; ^6^ Emergency Department, Línea de la Concepción Hospital, La Línea de la Concepción, Spain; ^7^ Department of Applied Biology-Nutrition, Institute of Bioengineering, Miguel Hernández University, Elche, Spain; ^8^ Group 36 - Nutrition and Physical Activity for Health, Alicante Institute of Health and Biomedical Research (ISABIAL), Alicante, Spain; ^9^ CIBER Fisiopatología de la Obesidad y Nutrición (CIBEROBN), Carlos III Health Institute (ISCIII), Madrid, Spain; ^10^ Nutrition and Physical Activity Research Group, Spanish Nutrition Society (SEÑ), Madrid, Spain; ^11^ Faculty of Psychology, University of Salamanca, Salamanca, Spain

**Keywords:** body composition, breast cancer, cancer survivors, exercise, hormonal response, melatonin, physical performance, quality-of-life

## Abstract

**Background:**

Breast cancer has a high prevalence in women during the last years of their life. Exercise is instrumental during this recovery period. Nevertheless, little is known about the effects of combining nutritional supplements with physical activity. Therefore, this study aims to examine the impact of melatonin in conjunction with physical activity in breast cancer survivors (BCS).

**Methods:**

Participants were postmenopausal women (60–75 years old) who had been diagnosed with stage I-III breast cancer 5 years ago and had received chemotherapy or radiotherapy. Participants were randomly assigned to two groups: experimental group (MEL) (*n* = 10), which received melatonin supplementation (6 mg/day), and the control group (CG) (*n* = 10), which received a placebo. Both groups followed an adapted physical activity program. After 10 weeks, body composition, physical condition, health-related quality of life and hormonal pattern were assessed in a randomized, single-blind, placebo-controlled trial (Clinical Trials.gov ID NCT06696378) following the Consolidated Standards of Reporting Trials. A Two-way repeated-measures analysis of variance (ANOVA) was used to examine the interaction effects (time × group) between MEL and CG. A significance level of *p* < 0.05 indicated a statistically significant difference.

**Results:**

After 10 weeks, both groups showed a non-significant decrease (*p* > 0.05) in fat mass. Both MEL and CG exhibited a significant reduction (*p* < 0.05) in the Borg Rating of Perceived Exertion (RPE) when comparing the beginning (T1) and end (T2) of the study Additionally, statistically significant differences (*p* = 0.018) were observed overtime between T1 and T2 in the MEL and CG in RPE, with a moderate effect size (*η*^2^*p* = 0.347). On the other hand, the Quality-of-Life Questionnaire (four domains and total score) and Short Physical Performance Battery indicated no significant (*p* > 0.05) differences between MEL and CG. Finally, testosterone/cortisol ratio decreased in both groups at the end of intervention, but the difference was not statistically significant (*p* > 0.05).

**Conclusions:**

Melatonin supplementation (6 mg/day) for 10 weeks, combined with a physical activity program, had not significant (*p* > 0.05) effects on anthropometry, physical condition, health-related quality of life and hormonal response compared to the placebo group. Our findings suggest no clear effect of melatonin in post-treatment for BCS in the mentioned parameters. Further clinicals trials are recommended to establish definitive recommendations for physical activity and melatonin supplementation in BCS.

**Clinical Trial Registration:**

Clinical Trials.gov, identifier (NCT06696378).

## 1 Introduction

In 2022, 2.3 million women were diagnosed with breast cancer (BC) and 670,000 died worldwide from the disease. BC affects women of all ages globally after puberty, with its incidence increasing with age (1). Scientific advances in the last decades have improved our understanding of BC biology, leading to the development of new drugs, the identification of prognostic factors, and personalized therapies, thereby optimizing treatment protocols. These advancements have collectively contributed to an increase in average overall survival rates (2).

However, survival presents a dual challenge for breast cancer survivors (BCS): they must work to restore their psychosocial and physical well-being while managing symptoms and treatment side effects. The disease itself, along with adjuvant chemotherapy and radiotherapy, is associated with serious complications such as muscle wasting and weakness (3). These adverse effects stem not only from the therapies (chemotherapy and radiotherapy), but also from physical inactivity, which is common among cancer patients and exacerbates the decline in physical function, aerobic capacity, and quality of life (QoL) (4, 5). Therefore, although BCS are now living longer, disease burden and treatment-related toxicities continue to impair their QoL and overall well-being.

Approximately 50% of BC cases are diagnosed in women aged up to 65 years, and over 30% in women aged 70 or older (6). With increasing life expectancy, projections indicate that by 2035, 60% of new BC cases will be diagnosed in patients aged 70 and older (7). BC in women over 70 presents specific clinical and biological characteristics that require tailored diagnostic and therapeutic approaches (8). Moreover, treating BC in older patients remains challenging, as older women are less likely to receive standard treatment for the disease (9). This clinical challenge underscores the need of pharmacological adjuvant treatment strategies to help manage this patient group by modulating BC and reducing treatment side effects (10). Among these strategies, therapeutic physical exercise (PE) and nutritional interventions, particularly nutritional supplementation, have shown promising results (11).

PE plays a key role in mitigating many of the adverse effects of BC therapy, enhancing physical performance, and reducing fatigue in BCS (4, 5). PE may help reduce chronic inflammation and stimulate the proliferation and differentiation of muscle stem cells (satellite cells) as part of the adaptive response to exercise, helping in recovery from muscle wasting and weakness (12). Additionally, PE supports mental and social well-being in BCS, alleviating symptoms associated with the disease or its treatment (13). However, single exercise sessions are unlikely to produce significant adaptive changes; instead structured PE programs over extended periods of time are required (11).

Melatonin (N-acetyl-5-methoxytryptamine) is a hormone synthesized by the pineal gland and other tissues or organs such as the retina, gastrointestinal tract and skin (14–16). Endogenous melatonin is synthesized from tryptophan, which is converted into serotonin before undergoing acetylation and methylation to form melatonin (17). Melatonin plays a fundamental role in regulating circadian rhythm (18, 19) and contributes to various gastrointestinal functions such as mucosal protection and gastrin release (15, 20). Moreover, melatonin exhibits anti-inflammatory (21), antioxidant (22, 23), and immunoregulatory (24) properties, acting as an antitumor agent (25, 26) and free radical scavenger (27, 28).

In cancer treatment, melatonin provides several benefits, including mitigating the toxic effects of chemotherapy (29) and radiotherapy (30). Specifically in BC, melatonin has been associated with inhibiting cell division and stimulating cell necrosis (31). Additionally, melatonin has demonstrated antiestrogenic properties (32, 33), due to its ability to reduce steroid production in the gonads; decreasing the synthesis of enzymes involved in the conversion of androgens to estrogens and binding directly to estrogen receptors (34, 35). Likewise, the role of melatonin in cancer extends to improving the QoL of patients with this pathology (36), particularly by addressing one of the main consequences of chemotherapy and radiotherapy: circadian rhythm disruption (37). Supplementing with exogenous melatonin can help reduce treatment-related side effects, including fatigue and sleep disturbances, thereby optimizing overall treatment outcomes (38).

Since cancer is strongly associated with aging, demographic trends suggests that the incidence of epithelial neoplasms will increase in the coming decades (1). BC is currently the leading cause of cancer-related death among women, with age being the primary risk factor for its development (39). Furthermore, the biological and clinical changes associated to aging influence cancer progression and treatment (9). Since most BC treatment and prevention guidelines for older adults are based on studies conducted on younger populations, research specifically targeting older adults would be highly beneficial (40). PE and appropriate nutritional supplementation are fundamental to both individual and community health. Therefore, this study aims to examine the effects of a 10-week melatonin supplementation combined with a PE program, on physical and anthropometric parameters, QoL, and hormonal response in elderly female BCS.

## 2 Materials and methods

### 2.1 Ethical considerations

The Ethics Committee of the University of Leon (Spain) (Supplementary Appendix A) approved the trial protocol (Approval Code ETICA-ULE-11-2024). The study was conducted in accordance with the principles the Declaration of Helsinki and its Fortaleza update (2013) (World Medical Association, 2013) (41). All participants signed an informed written consent before enrolling in the study, and each patient retained a copy of the signed consent form. Our study, “Melatonin Supplementation and Exercise Program in Breast Cancer Women (MEXBSO)”, was registered and approved by Clinical Trials.gov on November 18, 2024 (NCT06696378).

### 2.2 Experimental design

A randomized, single-blind, placebo-controlled trial was conducted to assess the effects of 10-weeks of melatonin supplementation on body composition, physical condition, health-related quality of life (HRQoL), and hormonal response in BCS. Participants were randomly assigned to either the experimental group (*n* = 10), which received melatonin supplementation (6 mg/day), or the control group (*n* = 10), which received a placebo. Randomization followed the Consolidated Standards of Reporting Trials (CONSORT) guidelines for parallel-group randomized trials (Supplementary Appendix B) (42). The sample size was determined using G*Power 3.1 software (43). A power analysis (1 − *β* error probability) indicated that a sample size of 18 participants would be sufficient to detect a difference of at least 1% in the hormonal biomarkers (testosterone or cortisol). The statistical power was set at 0.95, with a estimated effect size of 0.8 (44).

The study included two days of testing for each BCS, conducted under identical conditions and separated by a 10-week interval period (December 2, 3, and 4, 2024 and February 10, 11, and 12, 2025). Tests were standardized to begin at 9:00 a.m. both days to minimize circadian influences, with all procedures performed according to the same protocol, sequence, and timing. Anthropometric parameters, physical activity, quality of life and hormonal response were evaluated. Five days before the study commenced, all participants received instructions regarding the exercise protocols, melatonin supplementation (only in the supplemented group), and the type of tests assessed in the study to ensure proper progression. Additionally, all participants' medical records were reviewed.

### 2.3 Study population

Recruitment took place in November 2024 with convenience sampling through announcements posted on the bulletin boards at the Faculty of Health Sciences of the University of Valladolid, and three municipal sports centers in Soria (Spain). Adult BCS who responded to the invitations received a phone call with detailed information about the study, including its objectives, methodology, benefits, and potential risks. The 10-week intervention period lasted until March 2025.

Eligible participants were postmenopausal women aged 60 and older with a history of ductal carcinoma *in situ*, lobular carcinoma *in situ*, or stage I to III breast cancer, diagnosed at least five years prior, and who had received oncological treatment. Two study investigators collected specific data and clinical characteristics related to BC, 5 days before intervention by reviewing medical records. At the time of recruitment, participants were not undergoing chemotherapy, radiotherapy or hormone therapy and had an Eastern Cooperative Oncology Group (ECOG) performance status score of ≤1 (45). The two groups exhibited a close homology. To randomly assign participants to the two groups, block randomization was used. Each BCS has an equal probability of being assigned to either ML or CG.

The exclusion criteria were as follows: Stage IV breast cancer or systemic recurrences, previous malignancies other than BC, autoimmune diseases, use of oral melatonin supplements in the last 30 days, and any condition that physician deemed a contraindication to physical activity. Additionally, women who had engaged in regular PE for at least 30 min once a week in the three months preceding the study, were also excluded.

**Figure 1:**
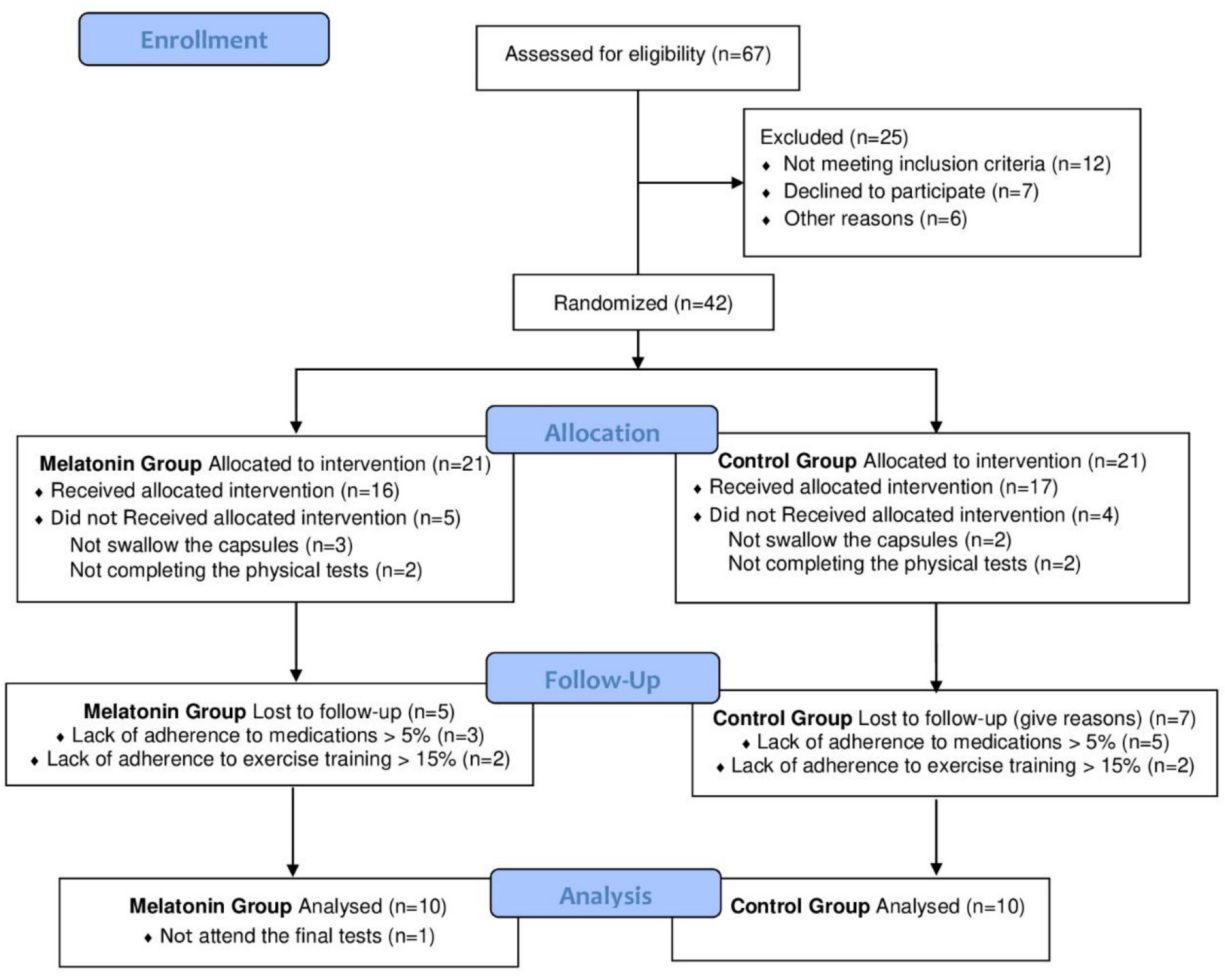
CONSORT flowchart (42) of participants recruited for the study.

A total of 67 BCS were recruited for initial assessment to form part of the study's participant sample. However, 25 women were excluded: 12 did not meet the inclusion/exclusion criteria, 7 declined to participate, and 6 were excluded for other reasons. Forty-two women met the inclusion criteria, were contacted, and received a detailed explanation of the project's objectives and methodology. These 42 participants were randomly assigned to two groups: 21 women in MEL and 21 in the CG. In the MEL group, 16 BCS received melatonin supplementation; however, 5 participants were lost to follow-up, including 3 due to lack of adherence to medications and 2 due to lack of adherence to exercise training. At the final stage, 1 BCS was excluded due to family-related issues preventing attendance at the final test. Therefore, the final MEL group consisted of 10 BCS. In the CG, 17 women received allocated intervention, but 4 BCS did not comply: 2 did not swallow the capsules, and 2 did not completed the physical tests. During follow-up, 7 BCS were excluded due to lack of adherence to medication (*n* = 5) or physical training (*n* = 2), resulting 10 women being included in the CG (Figure 1).

### 2.4 Randomization and blinding

Following baseline evaluation, participants were randomly assigned in a 1:1 ratio to receive either melatonin or placebo once daily for 10 weeks. Assignment to MEL (6 mg/day) or CG (placebo) was carried out using the Research Randomizer software (version 4.0) (46). Participants were blinded to their treatment allocation, ensuring the single-blind design of the study. The blinding index was calculated following the questionnaire developed by Hróbjartsson et al. (47) which assesses the effectiveness of blinding in clinical trials.

### 2.5 Melatonin supplementation

Melatonin supplements were provided from Nutrifoods® Laboratories (Barcelona, Spain) through the Magistral Formulation Laboratory in Soria (Spain), and prepared in accordance with the standards of the Royal Spanish Pharmacopoeia (Ministry of Health, Government of Spain). The melatonin technical data sheet (Reference No.: DIE-134) certified its composition and purity. Each capsule contained 6 mg of melatonin, 3 mg of colorant E-102/110, maltodextrin as excipient, and magnesium stearate as anti-caking agent, encapsulated in blue No. 4 capsules. Identical blue capsules containing only maltodextrin were used as placebo.

A study investigator was responsible for capsule distribution and randomization. Participants received a 10-week supply at the baseline visit, five days before the intervention began. They were instructed to take the assigned capsule (melatonin or placebo) daily at 9 p.m. or 30 min before bedtime. Participants were asked to record any missed doses in medication diaries. In the end, no missed doses were reported.

According to the MELODY clinical trial report (NCT01355523) a daily intake of 6 mg of melatonin is considered safe for human use over 12-week period (48–50). Ma et al. (29) reported that melatonin exhibits protective effects against chemotherapy-induced toxicity. However, melatonin-related side effects are rare, with 1–10 patients out of 1,000 experiencing short-term symptoms associated with daytime dosing (51). Therefore, it is essential to inform the BCS participating in our study about the possible occurrence of minor, manageable adverse effects (48–50), such as irritability, nervousness, restlessness, insomnia, abnormal dreams, anxiety, migraine, lethargy, psychomotor hyperactivity, dizziness, drowsiness, and hypertension (52).

The study team conducted monthly follow-up calls to monitor medication adherence, usage, and side effects. Any adverse events were documented according to the criteria established by the Spanish Agency of Medicines (Ministry of Health, Government of Spain), classifying events as mild, moderate, severe, or fatal. The study protocol stipulated that the trial would be discontinued if any moderate, severe, or fatal adverse event potentially related to the intervention occurred. Weekly telephone reminders were provided, and participants who adhere to less than 95% of the supplementation regimen were excluded from the study (Figure 1).

### 2.6 Exercise training

The PE program was designed following the FITT (Frequency; Intensity; Type; Time) guidelines recommended by the American College of Sports Medicine (ACSM) for BCS. The ACSM provides evidence-based physical activity recommendations, safety guidelines, and associated benefits for cancer prevention and survival (53). Our PE training program was designed and supervised by a physician and a physiotherapist from the research team. PE intensity was progressively adjusted according to the Borg Rating of Perceived Exertion (RPE) scale, specifically the Category Ratio-10 (CR-10) (54). RPE is a recognized marker of intensity and of homeostatic disturbance during exercise (55), allowing for the prescription of individualized exercise levels (56). We defined RPE ≤5 as a light intensity, RPE 6–8 as moderate intensity, RPE 7–9 as vigorous intensity, and RPE 15 as the threshold between heavy and severe intensity. Participants who missed more than 15% of the sessions were excluded from the study.

Participants engaged in the PE training program twice weekly for 10 weeks. Each 60 min session included: 10-min warm-up, 40 min main workout (5 min of balance exercises at RPE ≤5, 25 min of strength exercises at RPE 6–8, and 10 min of aerobic endurance training at RPE 6–8 or RPE 7–9), followed by a 10-min cooldown (RPE ≤5). The PE regimen was structured in three phases: (i) Phase 1 (Weeks 1–3): Resistance exercises were performed using only body weight; (ii) Phase 2 (Weeks 4–7): Resistance exercises incorporated a green elastic band (4–10 lbs or 2–4.5 kg), allowing for 10–12 repetitions per exercise; (iii) Phase 3 (Weeks 8–10): A blue elastic band (11–15 lbs or 5–7 kg) was used for resistance exercises, performed at a level allowing for 10–12 repetitions.

### 2.7 Anthropometric parameters

A single observer assessed body mass, fat mass, and body mass index (BMI) using resistance and reactance measurements obtained with a bioimpedance analyzer (BC-730; Tanita®, Japan). A constant alternating current of 800 µA at a frequency of 50 kHz was used (57). Bioimpedance analysis was used as it is considered an accurate method to evaluating fat mass (58). Tall was measured with a stadiometer. Anthropometric parameters were evaluated at the beginning (T1) and end (T2) of the study.

### 2.8 Physical tests

Physical abilities were measured by performing the following tests:

#### 2.8.1 Short physical performance battery

The Short Physical Performance Battery (SPPB) was used to assess physical performance at both study points, T1 and T2. SPPB consists of 3 tests: balance, gait speed, leg strength. The total score determines the patient's overall level of physical fitness (59). To minimize participant fatigue, the SPPB was performed in the following sequence: (i) Balance test in three positions: feet together, semi-tandem and tandem; (ii) 4-meter walking speed test to evaluate gait speed; (iii) Chair stand test (5 repetitions) to assess lower body strength. Scores range from 0 to 12, with higher scores indicating better function. A score below 10 is often associated with mobility limitations and increased risk of negative outcomes such as frailty and falls. The SPPB total score ranges from 0 (worst performance) to 12 points (best performance) and categorically evaluates performance in the tests using three or four classes of scores: three classes: 0–6 points (poor performance), 7–9 points (moderate performance), and 10–12 points (good performance) (59).

#### 2.8.2 Determination of perceived exertion

In sports, health, and exercise testing, the RPE measured using the Borg scale (54), is a quantitative tool for assessing exertion levels during physical activity. Numerical interpretation of the Borg CR-10 RPE scale, along with its validity, is as follows: 0-no exertion, 0.5-noticeable; 1-very light; 2-light; 3-moderate; 4-somewhat difficult; 5-difficult; 6 or 7-very difficult; 8 or 9-almost maximal; 10-maximal. Before blood collection, participants were asked to rate their perceived muscle discomfort at both T1 and T2 of the study using the validated Borg CR-10 scale for RPE (54).

### 2.9 Quality of life

QoL was assessed at T1 and T2 using the Spanish version of World Health Organization Quality of Life survey, brief version (WHOQOL-BREF) (60). WHOQOL-BREF is comprised of 24 items covering four dimensions: physical and psychological health, social relationships, and environment. Higher scores indicate better QoL. Items are answered on a five-point scale, domain scores ranged from 4 to 20, with high scores representing higher QoL (60).

### 2.10 Blood sample collection

All participants visited the laboratory for blood collection at T1 and T2. Blood samples (10 ml each) were collected from the antecubital vein after overnight fasting. Blood samples collection was carried out and supervised by a nurse and a physician from the research team. Thirty min before the extraction, participants relaxed, and blood sample collection began at 9:00 a.m. The tubes were then centrifuged at 5,000× g for 10 min. The serum was collected in EDTA-containing tubes, aliquoted, and stored at −80°C. Samples were analyzed in a certified hospital laboratory of the Spanish Public Health System, located in University Health Complex of Soria.

#### 2.10.1 Hormone determination in peripheral blood

Total testosterone levels were assessed using ELISA (DRG Instruments GmbH, Marburg, Germany). Cortisol concentration was assessed by enzyme-linked fluorescence assay (ELFA) technology with ready-to-use reagents on a compact multiparametric immunoanalyzer Minividas® (Biomerieux, Marcy l'Etoile, France). All determinations were performed according to the manufacturer's protocols. Hormone determinations (total testosterone and cortisol) were conducted at two points in the study: T1 and T2.

### 2.11 Statistical analysis

Analyses were performed using STATA version 15.0 (StataCorp, College Station, TX, USA), SPSS software version 24.0 (SPSS, Inc., Chicago, IL, USA), and Microsoft Excel (Microsoft Excel Software version 19). Data are presented as means ± standard deviations. A *p*-value <0.05 was considered statistically significant. The Shapiro–Wilk test was used to assess a normal distribution. Since the data followed a normal distribution, parametric tests were applied.

Barlett and Levene's tests were used to assess the equality of variances. Intergroup comparisons were conducted using one-way analysis of variance (ANOVA). Two-way repeated measures ANOVA was performed to examine the interaction effects (time × group) between the groups (MEL and CG) for the following variables: hormonal responses (testosterone, cortisol, and testosterone/cortisol ratio), physical performance (RPE and SPPB), anthropometric values (Body mass, BMI and fat mass), and WHOQOL-BREF domains (physical and psychological health, social relationships, and environment).

Within each group, differences between T1 and T2 were analyzed using Student's *t*-tests for parametric data. Effect sizes were calculated using a partial eta-square (*η*^2^*p*) (61). Since this measure tends to overestimate effect sizes; interpretations were made with caution, based on the following thresholds: no effect (*η*^2^*p* < 0.05), minimal effect (0.05 < *η*^2^*p* < 0.26), moderate effect (0.26 < *η*^2^*p* < 0.64), and strong effect (*η*^2^*p* ≥ 0.64).

## 3 Results

The study participants were Spanish women aged 60–75. Table 1 presents the participants' baseline characteristics. The body mass index was within the normal weight range (20–25 kg/m^2^) in the MEL group and slightly overweight (>25 kg/m^2^) in the CG group, although the differences were not significant. Smoking habits are one of the most determining variables in the development of breast cancer. There was only one smoker in both groups, while the rest of the participants were equally distributed between women who had quit smoking (non-smoker) and those who had never smoked (never smoker). In this regard, both groups were quite homogeneous. Regarding allergies, both groups also presented homogeneity, with the majority being women without allergies (70%–80%). This ensured that they could adhere to the physical activity plan without experiencing respiratory problems during the exercises. In fact, 20% of the women who reported having allergies did not experience any allergic crises during the intervention protocol, maintaining proper respiratory function. Regarding other vital signs, both groups had slightly elevated blood pressure values (144.22/86.22 mmHg in CG and 146.29/77.71 mmHg in MEL) and a resting heart rate in accordance with their age range (60–100 beats per min). Temperature data indicated that they did not suffer from any infections causing fever at the time of recruitment, and their oxygen saturation was within the correct range (95%–98%) for their age group. All these values ensured that the participants could successfully complete the physical activity protocol and that the results obtained were comparable between the two groups (MEL and CG), as well as within the same group between the beginning (T1) and end (T2) of the study (Table 1).

**Table 1:** Baseline characteristics of the participants.

Characteristics	Control (*n* = 10)	Melatonin (*n* = 10)
Gender, *n* (%)		
Female	10 (100.0)	10 (100.0)
Age (years), mean (SD)	65.5 (4.52)	66.3 (4.66)
Nationality, *n* (%)		
Spanish	10 (100.0)	10 (100.0)
Body mass index (BMI), mean (SD)^a^	25.83 (2.67)	24.07 (3.85)
Smoker, *n* (%)	1 (10.0)	1 (10.0)
Non-Smoker	6 (60.0)	4 (40.0)
Never Smoker	3 (30.0)	5 (50.0)
Known allergies, *n* (%)		
Yes	2 (20.0)	3 (30.0)
No	8 (80.0)	7 (70.0)
Vital signs, mean (SD)		
Blood pressure		
SBP (mmHg)	144.22 (18.57)	146.29 (16.51)
DBT (mmHg)	86.22 (10.78)	77.71 (8.26)
Heart rate (bpm)	73.67 (11.14)	79.29 (17.61)
Temperature (°C)	35.8 (0.5)	35.9 (0.4)
Oxygen saturation (%)	96.9 (1.7)	96.4 (1.6)

^a^ Results obtained according to Spanish Obesity Society (SEEDO) criteria. Non-significant differences between both groups assessed using one-way ANOVA were found.

Values are expressed as mean (SD) for quantitative variables and as frequency (percentage) for categorical variables.

bpm, beats per minute; °C, degrees Celsius; DBT, diastolic blood pressure; mmHg, millimeters of mercury; SBP, systolic blood pressure; SD, standard deviation.

All patients (*n* = 20) underwent surgery. The most common procedure was lumpectomy with sentinel lymph node removal (CG = 4; MEL = 3). Seven BCS received radiotherapy (3 in MEL, 4 in CG), 8 underwent chemotherapy (5 in MEL, 3 in CG), and 2 received both treatments (1 in MEL, 1 in CG). Five participants received hormone therapy (3 in MEL, 2 in CG). The predominant stage in Stage I–III BC was stage I in the CG (*n* = 5) and stage II in the MEL group (*n* = 5) (Table 2).

**Table 2:** Clinical characteristics of study participants.

Characteristics	Control (*n* = 10)	Melatonin (*n* = 10)
Type of surgery, *n* (%)	10 (100.0)	10 (100.0)
Mastectomy + axillary dissection ± SN	1 (10.0)	2 (20.0)
Mastectomy + SN	2 (20.0)	1 (10.0)
Lumpectomy + axillary dissection ± SN	2 (20.0)	3 (30.0)
Lumpectomy + SN	4 (40.0)	3 (30.0)
Lumpectomy converted to mastectomy + SN	1 (10.0)	0 (0.0)
Bilateral lumpectomy + axillary dissection + SN	0 (0.0)	1 (10.0)
Oncological treatment, *n* (%)		
None	2 (20.0)	1 (10.0)
Radiation	4 (40.0)	3 (30.0)
Chemotherapy	3 (30.0)	5 (50.0)
Radiation + Chemotherapy	1 (10.0)	1 (10.0)
Anti-hormone therapy, *n* (%)		
Yes	2 (20.0)	3 (30.0)
No	8 (80.0)	7 (70.0)
Cancer stage^a^, *n* (%)		
0	0 (0.0)	0 (0.0)
I	5 (50.0)	3 (20.0)
II	4 (40.0)	5 (50.0)
III	2 (10.0)	2 (30.0)
IV	0 (0.0)	0 (0.0)

^a^ According to the National Cancer Institute: 0, the main tumor cannot be found; I–IV refers to the size or extent of the main tumor. The higher numbers correspond to larger tumors. IV indicates that the tumor was extended into nearby tissues.

SN, sentinel node.

Table 3 presents the main anthropometric characteristics of participants. In the CG, average body mass slightly decreased at T2 (*p* > 0.05), likely due to a reduction in fat mass. Conversely, MEL showed an increase in body mass at the end of intervention (T2), despite a decrease in fat mass. However, no significant differences were found when comparing MEL vs. CG at either time point (T1 or T2). Furthermore, no significant differences (*p* > 0.05) were observed between T1 vs. T2 within either group (MEL or CG).

**Table 3:** Anthropometric parameters of participants recorded at the beginning (T1) and the end (T2) of the study.

Parameters	Time	Control (*n* = 10)	Melatonin (*n* = 10)	*p* (TXG)	*η* ^2^ *p*	Observed power
Body mass (kg)	T1	66.93 ± 9.52	60.38 ± 5.39	0.289	0.078	0.175
	T2	66.42 ± 9.43	61.91 ± 5.98			
BMI (kg/m^2^)	T1	25.99 ± 2.61	23.98 ± 3.87	0.774	0.007	0.057
	T2	25.87 ± 2.68	24.32 ± 3.54			
Fat mass (%)	T1	33.73 ± 5.54	31.03 ± 7.42	0.676	0.012	0.069
	T2	32.81 ± 4.19	29.61 ± 6.37			
Fat Mass (kg)	T1	22.96 ± 6.51	19.03 ± 6.17	0.713	0.010	0.063
	T2	22.14 ± 5.43	18.54 ± 5.09			

The data are expressed as mean ± standard deviation. *p*: Statistical differences by two-way ANOVA (time × group). No effect (*η*^2^*p* < 0.05), minimal effect (0.05 < *η*^2^*p* < 0.26), moderate effect (0.26 < *η*^2^*p* < 0.64), and strong effect (*η*^2^*p* ≥ 0.64).

BMI, body mass index; G, group; Kg, kilogram; m, meter T, time; *η*^2^*p*, effect size.

Table 4 presents the SPPB index values, which showed no significant differences (*p* > 0.05) between groups or across time points. Both groups exhibited a decrease in RPE when comparing T1 vs. T2. This change over time was statistically significant (*p* = 0.018), with moderate effect size (*η*^2^*p* = 0.347) and a statistical power of 0.642. These results suggest similar reduction in perceived fatigue at T2 in both groups.

**Table 4:** Physical activity parameters of participants recorded at the beginning (T1) and at the end (T2) of the study.

Parameters	Time	Control (*n* = 10)	Melatonin (*n* = 10)	*P* (TXG)	*η*^2^p	Observed power
SPPB index	T1	11.12 ± 0.79	11.40 ± 0.54	0.852	0.004	0.056
	T2	11.34 ± 0.49	11.64 ± 0.52			
Borg CR-10	T1	7.15 ± 1.23	7.26 ± 1.65	**0**.**018**	0.347	0.642
	T2	5.89 ± 1.14*	5.77 ± 1.21*			

* and bold font indicates significant differences comparing T1 vs. T2 by two-way ANOVA (time × group).

Data are expressed as mean ± standard deviation. No effect (*η*^2^*p* < 0.05), minimal effect (0.05 < *η*^2^*p* < 0.26), moderate effect (0.26 < *η*^2^*p* < 0.64), and strong effect (*η*^2^*p* ≥ 0.64).

Borg CR-10, scale for rating perceived exertion; G, group; SPPB, short physical performance battery; T, time; *η*^2^*p*, effect size.

Table 5 presents the results of the four domains of the WHOQOL-BREF questionnaire and the total score obtained. Physical health scores did not significantly increase (*p* > 0.05) in the CG, nor were any significant changes observed in MEL (*p* > 0.05). The CG maintained a constant score in the psychological health domain, while MEL showed a decrease, though not significantly (*p* > 0.05). In the social relationship's domain, both groups exhibited a decrease in average scores, with no significant differences (*p* > 0.05) between them. Similarly, the environment domain remained relatively constant in CG but decreased in MEL, although this difference was not significant (*p* > 0.05). Regarding the total score, an increase (indicating improvement) was observed in CG, while a decrease was noticed in MEL. However, these differences were not statistically significant (*p* = 0.183).

**Table 5:** Scores of total and particular domains of WHOQOL-BREF (60) in women participating in the study.

Test	Time	Control (*n* = 10)	Melatonin (*n* = 10)	*p* (TXG)	*η* ^2^ *p*	Observed power
Physical health	T1	14.71 ± 1.79	15.47 ± 1.79	0.547	0.026	0.091
	T2	15.47 ± 1.77	15.41 ± 1.87			
Psychological	T1	15.29 ± 1.78	15.88 ± 2.73	0.242	0.097	0.204
	T2	15.29 ± 2.13	14.61 ± 1.56			
Social relationships	T1	15.57 ± 2.51	15.46 ± 2.57	0.829	0.004	0.056
	T2	13.82 ± 2.78	13.28 ± 1.96			
Environment	T1	15.09 ± 2.52	16.38 ± 1.83	0.105	0.177	0.362
	T2	15.97 ± 2.13	15.67 ± 1.68			
TOTAL Score	T1	98.83 ± 8.93	102.96 ± 12.69	0.183	0.119	0.258
	T2	100.27 ± 9.88	98.38 ± 8.65			

The data are expressed as mean ± standard deviation. p: *p* value by two-way ANOVA (time × group). No effect (*η*^2^*p* < 0.05), minimal effect (0.05 < *η*^2^*p* < 0.26), moderate effect (0.26 < *η*^2^*p* < 0.64), and strong effect (*η*^2^*p* ≥ 0.64).

Tests were passed at the beginning (T1) and at the end (T2) of intervention.

G, group; T, time; *η*^2^*p*, effect size.

Table 6 presents the hormonal response data, based on testosterone and cortisol levels. No significant (*p* > 0.05) differences were observed in hormonal patterns within either group or between the two groups.

**Table 6:** Hormonal pattern of women at the beginning (T1) and at the end (T2) of intervention.

Hormones (units) [reference range]	Time	Control (*n* = 10)	Melatonin (*n* = 10)	*p* (TXG)	*η* ^2^ *p*	Observed power
Cortisol (µg/dl) [5–25]	T1	15.98 ± 3.81	16.88 ± 2.47	0.987	0.000	0.051
	T2	17.87 ± 4.57	18.75 ± 3.73			
Testosterone (ng/ml) [0.06–0.82]	T1	0.45 ± 0.20	0.54 ± 0.12	0.298	0.073	0.172
	T2	0.37 ± 0.22	0.42 ± 0.09			
Ratio TT/C	T1	2.49 ± 0.95	2.92 ± 0.66	0.725	0.008	0.065
	T2	2.21 ± 1.10	2.43 ± 0.63			

The data are expressed as mean ± standard deviation. *p*: *p* value by two-way ANOVA (time × group). No effect (*η*^2^*p* < 0.05), minimal effect (0.05 < *η*^2^*p* < 0.26), moderate effect (0.26 < *η*^2^*p* < 0.64), and strong effect (*η*^2^*p* ≥ 0.64).

C, cortisol; G, group; T, time; TT, testosterone; *η*^2^*p*, effect size.

Blinding index ranged from 1 (none of the subjects knew to which group they were assigned) to 0 (all subjects knew to which group they were assigned). Values above 0.5 indicated that the blinding was successful (47). The blinding index at T2 was 0.86, with five participants stating that they knew which group they belonged to, although only three BCS were correct. This suggested that the blinding process was successful (Results not reported in table).

## 4 Discussion

This study investigated the effects of 10 weeks of melatonin supplementation combined with a PE program on body composition, physical condition, HRQoL, and hormonal responses in BCS. After the 10-week intervention, all measured parameters were modified in both MEL and CG. However, no significant differences (*p* > 0.05) were found between the groups, suggesting that the observed changes were likely due to the PE program. While previous research has highlighted the potential benefits of melatonin in managing certain aspects of BC (31, 62), the results of the present report indicate that melatonin supplementation has not a clear effect on the parameters examined in this study. No adverse effects related to the PE program or melatonin supplementation were reported. The only notable observation was an increase in BMI in the MEL group at the end of intervention, likely due to an increase in muscle mass. The results obtained in the different tests, suggest that melatonin does not appear to affect the measured parameters. Additionally, the hormonal pattern observed suggests that the stress associated to exercise remained constant through the study, with no influence of melatonin.

BC is a prevalent disease among older women, with approximately one-third of diagnoses occurring in women aged 60 and over (63). In addition, women in this age group have a 1 in 15 chance of developing additional tumors. With the projected growth of the aging population, these figures are expected to rise in the coming years (64). Treating BC in older women presents a therapeutic challenge, as BC-related mortality increases with age (9). Given the variability in symptoms presentation across different BC stages, this study focused on women aged 60–75 years with a history of BC stages I, II, and III. Demonstrating a positive response to the combined melatonin and PE intervention in this population could enhance the clinical applicability of the findings. Although survival was not an outcome assessed in this study, long-term survival analysis between the 2 groups could offer valuable insights into survival rates for BCS, potentially impacting clinical practice. Importantly, melatonin supplementation (6 mg/day) was well-tolerated, consistent with reports indicating that doses of up to 20 mg/day are generally safe (65, 66). Melatonin has also demonstrated protective effects against toxicity associated with oncologic treatments (29), mitigating adverse effects such as thrombocytopenia, neurotoxicity, or cardiotoxicity, among others (65, 66).

Our study found no significant differences in anthropometric parameters between MEL and CG. This may be due to the melatonin dosage, which seems to be insufficient to induce changes in body composition. However, studies in young obese and diabetic Zucker rats (an experimental model of metabolic syndrome) have shown that melatonin treatment (10 mg/kg/day) reduced white adipose tissue by promoting a partial conversion to brown fat, which allows fatty acid oxidation through thermogenic mechanisms (67). This effect of melatonin does not seem to be operative in humans. Consistent with this, melatonin supplementation did not reverse side effects such as weight loss or appetite changes associated with chemotherapy and radiotherapy in patients with advanced lung, gastrointestinal, or brain tumors (62). Furthermore, other clinical trials using melatonin dosages of 3 mg for 14 days in head and neck cancer (68), 6 mg for 3 months in BC patients (49), and 20 mg for 6 weeks in patients with various cancer types (69) did not assess body composition, making it challenging to determine the optimal dosage and duration for modulating anthropometric changes.

In the present study, as mentioned above, MEL and CG experienced a decrease in fat mass, but only MEL showed a non-significant increase (*p* > 0.05) in body mass at T2. A plausible explanation is that MEL experienced an increase in muscle mass, which would account for the observed rise in BMI at the end of the 10-week intervention. This increase in muscle mass is likely due to the melatonin-plus-exercise program. The combined aerobic and resistance exercise program implemented in this study is expected to improve both cardiovascular and muscle metabolism through muscle protein synthesis (70). This type of exercise program can help maintain muscle mass during weight loss, contributing to improved physical function and reduced frailty (71). Additionally, this exercise protocol could lower the risk of future cancer recurrence associated with overweight, as excess fat mass has been linked to increased estrogen production, which may promote the development and growth of hormone receptor-positive BC cells (72, 73).

For BCS, PE is widely recommended and has been associated with improvements in physical function (4), QoL (4) and increased survival (5). Physical parameters were evaluated using the SPPB index (59), which showed no significant differences (*p* > 0.05) between groups or across measurement times. This lack of significance is likely due to participants were non-frail (SPPB ≥10) before the study and remaining in this state at the end, suggesting a low likelihood of major health events such as hospital admission or exacerbation of chronic conditions. Moreover, patients enrolled in clinical trials are often highly selected for their robust physical health, making them potentially unrepresentative of the general geriatric population (74). Additionally, a recent study by Celorrio et al. (75) reported no direct relationship between melatonin supplementation and physical performance. The effects of melatonin on physical status could depend on factors like the type and duration of supplementation, or the type and duration of exercise, which may influence melatonin's impact on physical parameters (75).

The RPE CR-10 scale is used to assess effort and fatigue during physical activity (76). In this study, both groups recorded a significant decrease (*p* < 0.05) in RPE CR-10 values from T1 to T2, displaying significant (*p* = 0.018) trends over the course of the study. Melatonin supplementation (20 mg weekly) in phase IV cancer patients from a palliative care unit (77) or early-stage BC patients during and two weeks post-radiotherapy (78) did not improve fatigue, insomnia, or tiredness. However, a study by Dieli-Conwright et al. (79) found that a 16-week concurrent exercise program (aerobic and strength training) significantly reduced fatigue in BC patients. Similarly, our participants indirectly demonstrated reduced fatigue, as they were able to significantly increase exercise intensity. In this regard, elastic band exercises were progressively adjusted based on the RPE scale to reach a level of 10 (80), without inducing fatigue. These findings support the incorporation of exercise routines, which are associated with favorable survival trends, across various cancer populations (4). Given the clinical characteristics of the sample, acute physical exercise also increases oxidative stress (81). In this context, MEL reported lower RPE-CR-10 values, likely due to melatonin's antioxidant, anti-inflammatory, and circadian rhythm-regulating properties (82). However, these last points need further investigation. Lower RPE in BCS is associated with various clinical and HRQoL implications, including higher treatment adherence, lower risk of complications, and improved HRQoL. Fatigue, a common side effect, can limit physical activity, social participation, cognitive function, negatively impacting HRQoL, and the well-being of patients (83). RPE allows for prescribing individual exercise values (56) adjusted to FITT for each BCS, ensuring the absence of side effects or withdrawals from the study due to PE.

Although participants showed improvements in SPPB, RPE, and body composition, no changes were observed in the total score or dimensions (physical and psychological health, social relationships, and environment) of HRQoL, as measured by the WHOQOL-BREF questionnaire (60). This may be due to several factors, such as the impact of the disease on survivors (e.g., depression, low self-esteem, changes in physical conditions, loneliness, among others) at different stages (84). These factors can have long-term effects across all four HRQoL dimensions, often associated with disease progression or oncologic treatments received (85). In addition, our results might be influenced by the study's duration, the supplementation protocol, or the training protocol. It is possible that a 10-week intervention is too short to elicit measurable changes in HRQoL. Consistent with our findings, Seely et al. (86) also reported no QoL benefit in non-small cell lung cancer (NSCLC) patients receiving 20 mg of melatonin over two years. Similarly, the meta-analysis conducted by Fan et al. (87), which included 19 randomized controlled trials involving 2,101 cancer patients receiving melatonin supplementation, found no improvement in HRQoL. However, a systematic review by Fernández-Lázaro et al. (4) reported that PE programs significantly improved QoL compared to controls (no exercise). QoL has been evaluated using the European Organization for Research and Treatment of Cancer (EORTC) Quality of Life Questionnaires [Core-30 (QLQ-C30), Lung Cancer-13 (QLQ-LC13)] modules, the Functional Assessment of Cancer Therapy (FACT), and the Short Form-36 Health Survey (SF-36) (86). To our knowledge, this is the first study to use the WHOQOL-BREF questionnaire in a trial involving melatonin supplementation (60) and PE, although this HRQoL test is commonly applied in cancer-related research (88).

In healthy humans, melatonin and cortisol circadian rhythms display opposite phases: melatonin peaks at night when cortisol levels are low, and vice versa during the day (89). In contrast, abnormalities in the diurnal cortisol rhythm are common in cancer patients and have been associated with poorer survival in BC patients (37). Furthermore, cancer patients undergoing mixed treatments (chemotherapy and radiotherapy) or post-treatment often exhibit elevated cortisol levels, which can negatively affect QoL due to both the physical and emotional impact of the disease (90). In our study, cortisol levels increased slightly in both MEL and CG, suggesting that melatonin supplementation does not affect the MT1 melatonin receptor in the adrenal gland or inhibit adrenocorticotropic hormone (ACTH)-stimulated cortisol production (91). It remains unclear whether a higher dose or longer duration of treatment would have influenced daytime cortisol production. Studies on melatonin supplementation show no conclusive effects on plasma cortisol levels, with mixed findings indicating that melatonin either increases (92, 93), decreases (91, 94), or has no effect (95, 96). The high cortisol levels observed in our BCS, at T1 and T2 could be influenced by threat minimization and problem-focused coping strategies, which serve as mediating factors in adapting to BC (90). This triggers activation of the hypothalamic-pituitary-adrenal (HPA) axis, leading to cortisol secretion (91). When stress is persistent and uncontrollable, HPA axis activity decreases. However, when BCS stress has some controllable elements, such as fight-flight responses to BC, the HPA axis may be activated (97). The HPA axis plays an instrumental role in providing metabolic support, mobilizing energy to address cancer management and implications of oncological treatment (97). Cortisol is also released in response to physical and psychological stress, with significant increases following exercise due to training adaptations (98). Cortisol levels are generally highest in the morning, when blood was collected in this study, and lowest around midnight (89). All these factors may contribute to the observed increases in plasma cortisol levels. High cortisol concentrations are linked to chronic inflammation, which raises the risk of cellular epigenetic alterations (99). This explains why persistent chronic inflammation itself constitutes a risk factor for certain cancers (100), through activation of the NF-kB and STAT3 signaling pathways associated with tumor growth (101). Additionally, stress induces both indirect mechanisms (e.g., immune system inhibition) and direct mechanisms (e.g., binding of stress signaling molecules to cell receptors) that can promote cancer progression (102, 103). However, an increased cortisol response may help coping with stress during the daytime (104). Future studies should include more frequent, multi-day cortisol monitoring to better understand melatonin's true effects on cortisol.

Finally, testosterone levels tended to decrease substantially, though not significantly (*p* > 0.05), in both groups from T1 to T2, with no significant (*p* > 0.05) differences between-groups. The testosterone/cortisol ratio also decreased, but without significant differences (*p* > 0.05). The evidence regarding whether melatonin supplementation reduces testosterone levels is conflicting in animal models (105, 106), whereas studies in humans suggest no effect (75, 107). The notable decreases in testosterone observed in our study could be attributed to the continuous PE program. Testosterone levels are influence by exercise duration and intensity, which may disrupt the hypothalamic-pituitary-gonadal axis, leading to suppressed testosterone secretion (108). Testosterone plays an essential role in the physical and mental health, as well as in QoL of women, including BCS. The hormone is generally protective against BC by reducing tissue proliferation. However, testosterone can also be converted to estradiol (E2), which may increase BC risk due to stimulation of cell proliferation (109).

While exercise is beneficial, it can also exacerbate fatigue, increasing the perception of exertion, particularly in this population with significant impairment due to BC and BC treatments. This would directly affect the physical condition and QoL of BCS. Melatonin may help mitigate these adverse effects, providing a protective role against exercise-induced fatigue. Although the effects were not statistically significant, the qualitative differences in parameter responses between the MEL and CG groups could support the success of our intervention. Higher doses of melatonin or extended periods of exercise and supplementation may be required to achieve significant effects. However, our study uses three times the dose authorized by the Spanish Agency for Medicines and Health Products. Additionally, our structured exercise program was carefully tailored to the condition of our patients. It is also important to consider that some patients may be non-responders. Several factors could explain this lack of response, including underlying diseases, interactions with other medications, or individual variability in melatonin response. Furthermore, the dose or timing of administration may not be optimal for BCS, who are older adults. Age could influence the effect of melatonin through multiple mechanisms, including alterations in melatonin receptors, age-related hormonal changes, underlying health conditions, and the use of certain concomitant medications.

### 4.1 Limitations

The present study has several limitations, as it is an exploratory or pilot trial. The sample size is small (monocentric); a larger multicentric sample would provide more robust results. In addition, all QoL and RPE CR-10 data were self-reported. It is important to consider that BCS who voluntarily enroll in a structured physical activity program are likely to be more motivated and physically capable than the general BCS population, which may contribute to higher adherence to the exercise program. To mitigate the limitations of the self-reported data, we used broad and stringent inclusion and exclusion criteria, a simple randomization process, and blinded all BCS personnel to the intervention and placebo group assignments. The blinding rate was high (0.86). Furthermore, patient inclusion both before and during cancer treatment would be beneficial. It should be also considered that BC is classified into four subtypes based on clinicopathologic features defined by immunohistochemical expression of estrogen receptor (ER) or progesterone receptor (PR) and human epidermal growth factor receptor 2 (Her2): ER/PR+, Her2+; ER/PR+, Her2−; ER/PR−, Her2+; and ER/PR−, Her2− (110). Future subgroup analyses, including pre- or during-treatment assessments, may be crucial to understanding the benefits or limitations of melatonin supplementation in BC. Although individual patient characteristics vary, the results have limited generalizability. Finally, biochemical tests, particularly those measuring muscle damage markers, such as lactate dehydrogenase, creatine kinase, and myoglobin (111), were not included. Incorporating such analyses in future studies will be necessary.

### 4.2 Implications of the study and future directions

The findings of our study have important clinical implications for the management of anthropometric, physical, health-related QoL, and hormonal response parameters in BCS. They demonstrate that a combination of structured exercise and melatonin supplements can yield significant health benefits without causing harmful effects. This research could be of interest to oncologists, dietitians, physiotherapists, and physical trainers, as it represents an advancement in adjuvant strategies for BC. Therefore, implement nutritional support that helps BCS meet additional dietary needs and optimize their health and physical condition appears reasonable, aligning with the principles of rational nutrition. Regarding melatonin, achieving an efficient dose through diet alone is not feasible; although diet has an indirect effect, supplementation is necessary. While the results are promising, future studies should explore the long-term effects of this combined approach and investigate the underlying mechanisms. Nevertheless, melatonin might influence other parameters not assessed in this study, such as oxidative stress modulation, which plays a key role in cancer progression (62). Adjusting exercise intensity is also crucial, as some BC tumors metabolize lactate. This metabolite is a critical component of the tumor microenvironment and has emerged as a key factor influencing tumor growth, development and survival (112). In such cases, moderate-intensity exercises, rather than high-intensity exercises that produce more lactate (113), would be preferable. These considerations outline future research directions that we are exploring in our laboratory.

## 5 Conclusions

A 10-week supplementation of 6 mg/day of melatonin combined with a PE program did not influence anthropometric measure, physical parameters, HRQoL, and hormonal response compared to the placebo-supplemented group, which also participated in the PE program. These results further support the lack of a clear effect of melatonin in this context, suggesting that the observed modifications are likely due to the PE program itself. However, differences in biomarker responses between the MEL and CG groups may indicate some success of our melatonin supplementation protocol. These findings suggest a novel therapeutic strategy that could enhance health outcomes and QoL for individuals with BCS. Further research is needed to validate these results and explore the broader implications of this combined intervention.

## Data Availability

The original contributions presented in the study are included in the article/Supplementary Material, further inquiries can be directed to the corresponding authors.
