# Supplementary material for: Adjuvant melatonin therapy during exercise prescription in breast cancer survivors on physical and anthropometric parameters, quality of life, and hormonal response. A randomized controlled trial
Source: Front Sports Act Living. 2025 Jun 23;7:1594733. doi: 10.3389/fspor.2025.1594733 (PMC12229996; doi:10.3389/fspor.2025.1594733)

**Annex I.** The Ethics Committee of the University of Leon (Spain) approved the trial protocol as indicated by the Approval Code ETICA-ULE-11-2024.


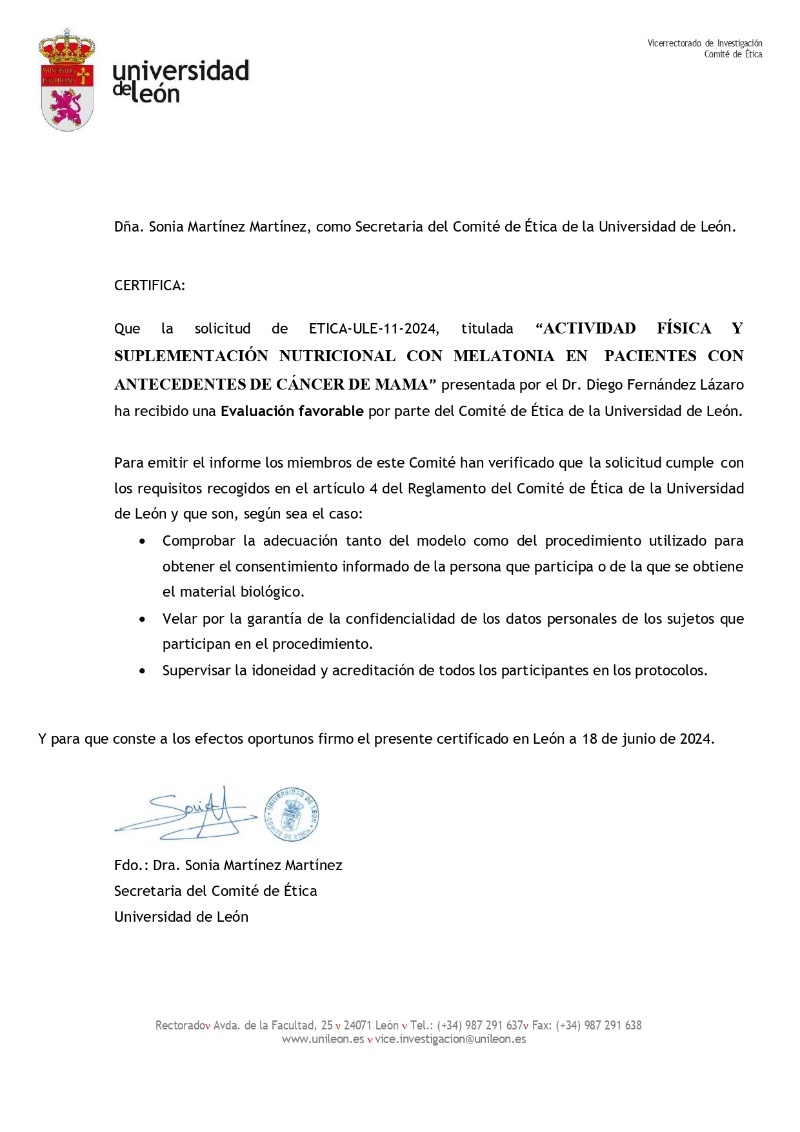

Supplement: Supplementary file 1 [file Table1.docx]
